# Cytoproliferative and Cytoprotective Effects of Striatisporolide A Isolated from Rhizomes of *Athyrium multidentatum* (Doell.) Ching on Human Umbilical Vein Endothelial Cells

**DOI:** 10.3390/molecules21101280

**Published:** 2016-09-24

**Authors:** Dong-Mei Liu, Ji-Wen Sheng, Si-Hong Wang, Wei-Fen Zhang, Wei Zhang, Dai-Juan Zhang

**Affiliations:** 1 Department of Pharmacy, Weifang Medical University, Weifang 261053, China; ldmwfmc@163.com (D.-M.L.); zwf2024@126.com (W.-F.Z.); WXY070604@126.com (D.-J.Z.); 2 Analysis and Test Center, Yianbian University, Yanji 133002, China; shwang@ybu.edu.cn; 3 Plastic Surgery Institute of Weifang Medical University, Weifang 261053, China; zhangwei@wfmc.edu.cn

**Keywords:** striatisporolide A, *Athyrium multidentatum* (Doell.) Ching, butenolides, cytoprotective effect, ROS, apoptosis

## Abstract

*Objectives*: The aim of this study was to investigate the proliferative and protective effects of striatisporolide A (SA) obtained from the rhizomes of *Athyrium multidentatum* (Doell.) Ching on human umbilical vein endothelial cells (HUVECs). *Methods*: Cell viability was measured by the MTT method. Cell apoptosis was determined by flow cytometry. Intracellular ROS was measured by the 2,7-dichlorodihydrofluorescein diacetate (DCFH-DA) fluorescent probe. *Results*: The viability rate in cells treated with 100 µM SA alone was increased to 128.72% ± 0.19% and showed a significant difference compared with the control group (*p* < 0.05). Meanwhile, SA augmented the cell viabilities in H_2_O_2_-treated HUVECs, and the cell viability was enhanced to 56.94% ± 0.13% (*p* < 0.01) when pre-incubated with 50 µM SA. The cell apoptosis rates were reduced to 2.17% ± 0.20% (*p* < 0.05) and 3.1% ± 0.34% (*p* < 0.01), respectively, after treatment with SA alone or SA/H_2_O_2_. SA inhibited the overproduction of reactive oxygen species (ROS) in HUVECs induced by H_2_O_2_ and the fluorescent intensity was abated to 9.47 ± 0.61 after pre-incubated with 100 μM SA. *Conclusion*s: The biological activities of SA were explored for the first time. Our results stated that SA exhibited significant cytoproliferative and minor cytoprotective effects on HUVECs. We presume that the mechanisms of the proliferation and protection actions of SA involve interference with the generation of ROS and the cell apoptosis. These findings provide a new perspective on the biological potential of butenolides.

## 1. Introduction

Natural products have been attracting continuous attention due to their unique structures and activities. Butenolides, a class of unsaturated lactones with a four-carbon 2(5H)/2(3H) furanone heterocyclic ring skeleton, are usually found in fungi, bacteria and gorgonians. Their saturated analogues serve as signaling molecules in bacteria to amplify spore formation in streptomycetes or induce metabolite production [1]. Some butenolides possess significant biological activities such as antitumor, antibacterial, antifungus, antioxidant, antiinflammatory, antiepileptic, plant growth regulating and herbicidal effects [2]. Caesalpinolide A and B, isolated from the marine creeper *Caesalpinia bonduc*, could inhibit MCF-7 breast cancer cell line cells with IC_50_ values of 12.8 and 6.1 μM [3]. Butenolide-containing dithiocarbamates exhibited broad spectrum anti-cancer activity against five human cancer cell lines, with an IC_50_ value of less than 30 μM [4]. The γ-alkylidene bicyclic butenolide cochinolide showed moderate antiviral activities against HSV-1 and -2 [5]. Protolichesterinic acid and lichesterinic acid from the lichen *C. islandica* (Iceland moss) had substantial antitrypanosomal activity against *T. b. brucei* [6]*.*

**Figure 1:**
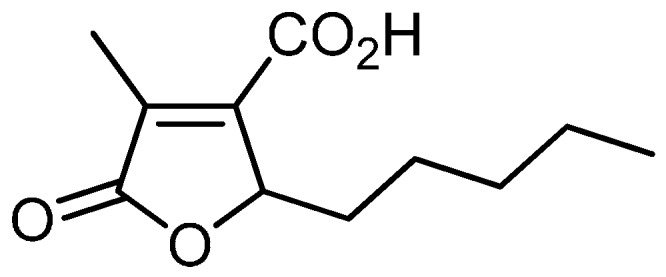
The chemical stucture of striatisporolide A (SA).

Striatisporolide A (SA) (Figure 1) is a simple butenolide derivative with a pleasant scent and limited distribution among a few bacterial strains, including *Penicillium striatisporum* and *Penicillium janthinellum* [7,8]. Inspiringly, SA has been synthesized starting from 2-alkylidene-3-methyl-succinimides via an acid catalyzed hydrolysis, esterification, OsO_4_-dihydroxylation and dehydrative cyclization pathway [9]. A recent study revealed that SA showed weak cytotoxic effects on A549 cells, with an IC_50_ value of 36.5 μM [8]. However, regardless of these results, there is very poor information about the pharmacological activities of SA.

*Athyrium multidentatum* (Doell.) Ching is a terrestrial fern in the family Athyriaceae, used as an largely unexplored folk medicine that has been used for thousands of years for conditions such as high blood pressure, anxiety and arthritis [10]. In our previous study, SA was obtained during the process of seeking antiaging substances from the rhizome of *Athyrium multidentatum* (Doell.) Ching. In order to explore the antiaging potential of SA, the in vitro cell viability, cell apoptosis and ROS levels were investigated employing HUVECs as a model system. 

## 2. Results and Discussion

### 2.1. Analysis of Cell Viability

**Figure 2:**
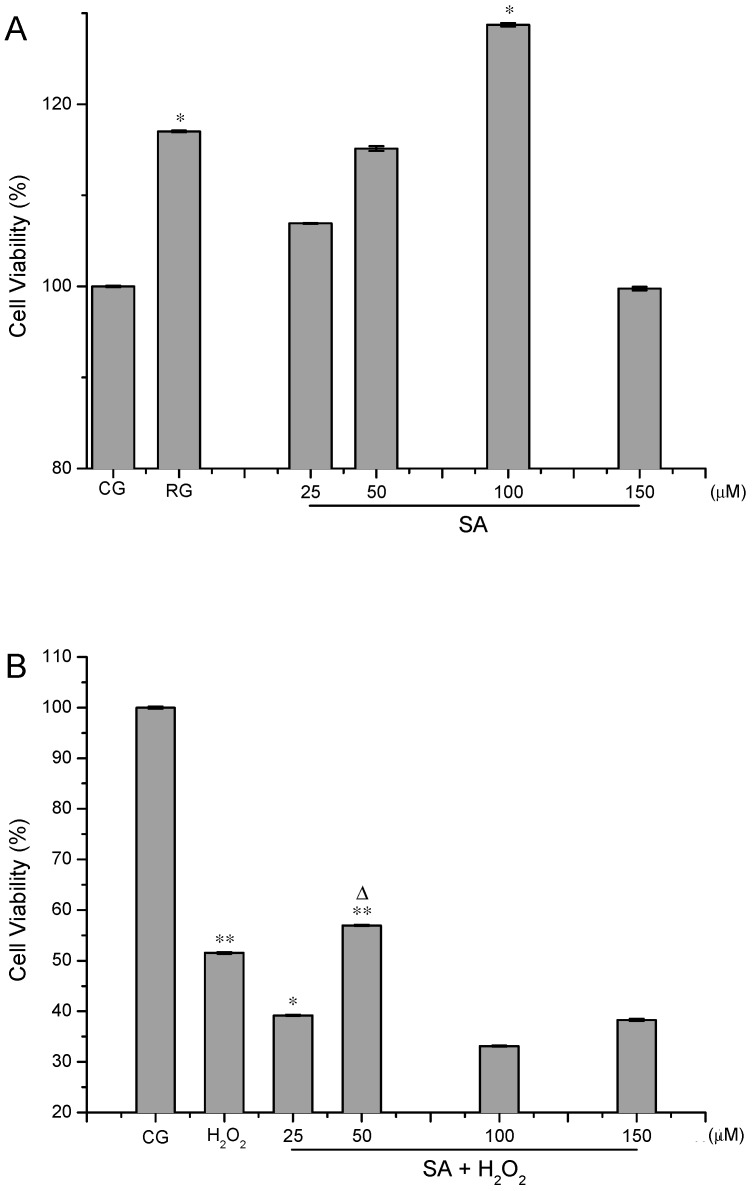
Effects of striatisporolide A (SA) on HUVECs untreated with H_2_O_2_ (**A**) and treated with H_2_O_2_ (**B**). CG represents as control group. RG represents as positive control group. H_2_O_2_ represents as the group treated with H_2_O_2_ alone. Values are means ±SD (*n* = 3). Compared with the control group (* *p* < 0.05, ** *p* < 0.01). Compared with the H_2_O_2_ group (^∆^ *p* < 0.05).

The proliferative and protective effects of SA on HUVECs are displayed in Figure 2A,B. Compared with the control group, the cell viabilities were over 100% after the cells were treated with SA alone and a dose-dependent effect was observed between 25 and 100 µM (Figure 2A). SA increased the cell viability to 128.72% ± 0.19% (*p* < 0.05) at the concentration of 100 µM, which was much stronger than resveratrol. However, a higher dose of SA (150 µM) led to decreased cell viability (99.76% ± 0.2%). This meant that HUVECs displayed the best viability when exposed to 100 µM SA. In addition, SA showed protective effects on HUVECs against H_2_O_2_-induced oxidative injury (Figure 2B). In comparison with the control group, the cell viabilities were strikingly minimized after the cells were exposed to 300 µM H_2_O_2_ (51.54% ± 0.20%, *p* < 0.01). After pre-incubation with 50 µM SA, the cell viability was enhanced to 56.94% ± 0.13% and showed a significant difference compared with the control group (*p* < 0.01). However, there was no dose-effect relationship between SA and cell viabilities in H_2_O_2_-treated cells. These results indicated that SA was more powerful at boosting cell proliferation than protecting the cells against H_2_O_2_-induced oxidative damage. Chemical molecules with proliferative and protective properties are beneficial for repairing and regenerating damaged tissues. Natural products with proliferation activity are very popular for their low toxicity and minor side effects. Heteropolysaccharide from the fruiting bodies of *Lactarius deliciosus* Gray could significantly promoted B cell and RAW264.7 cell proliferation at 5 μg/mL with proliferation rates of 124.73% and 138.01%, respectively [11]. A *Terminalia bellirica* extract with gallic acid as a major component was found to possess cytoprotective activity and could significantly boost human mesenchyamal stromal cell (hMSC) proliferation and cell attachment [12]. The proliferation and differentiation of hMSCs play an important role in tissue replacement and recovery in diseases including osteoporosis, heart disease, Parkinson’s disease and cartilage replacement [13]. The cytoprotective mechanism is associated with the activation or inhibition of certain signaling pathways. Microcystin-LR produced by blue-green algae could promote cell proliferation in vivo by activating the Akt/mTORC1/S6K1 pathway and inhibiting protein phosphatase 2A activity [14]. Resveratrol exerts protective effects against both acute and chronic kidney injuries through its antioxidant effects and ability to activate SIRT1 [15]. In order to explore the protective mechanism of SA, we have examined the protein levels of SIRT1 and p53 in HUVECs. However, SA showed no regulation activities on SIRT1 and p53. The cytoprotective mechanisms of SA was directly associated with its anti-apoptotic capacity and interference with ROS generation, which have been verified in the following assays. 

### 2.2. Cell Apoptosis Assays 

**Figure 3:**
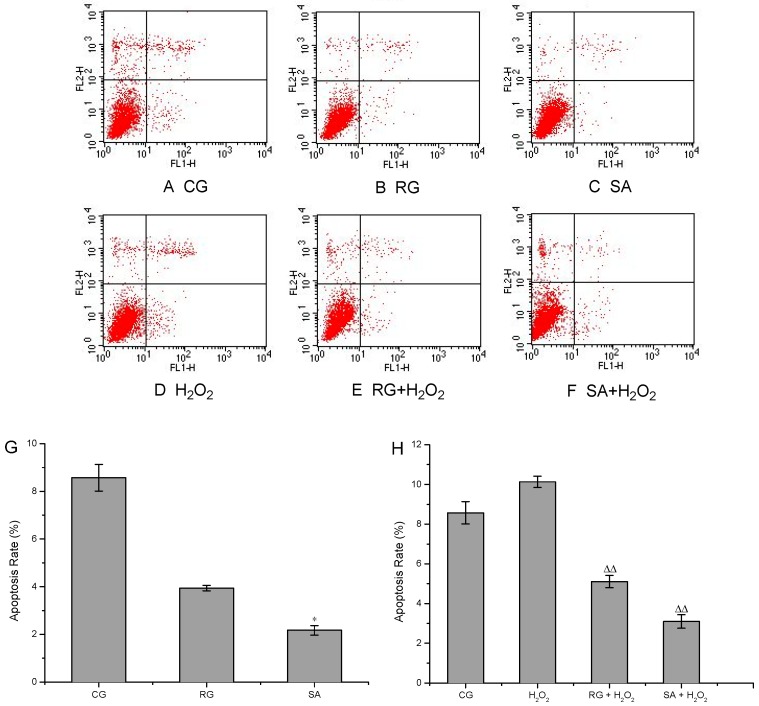
Apoptosis analysis of HUVECs in response to striatisporolide A (SA) by flow cytometry. **A**–**C**: Flow cytometric analysis of cells untreated with H_2_O_2_. **D**–**F**: Flow cytometric analysis of cells treated with H_2_O_2_. **G**: Apoptosis rate in cells untreated with H_2_O_2_. **H**: Apoptosis rate in cells treated with H_2_O_2_. CG represents as control group. RG represents as positive control group. H_2_O_2_ represents as the group treated with H_2_O_2_ alone. Values are means ± SD (*n* = 3). Compared with the control group (* *p* < 0.05). Compared with the H_2_O_2_ group (^∆∆^ *p* < 0.01).

Cells were treated with 100 µM SA in the apoptosis assays for its marked proliferation promoting effect on HUVECs at this concentration. As presented in Figure 3H, the apoptosis rate was increased to 10.13% ± 0.28% after the cells were treated with H_2_O_2_, which was much higher than that in the control group (8.57% ± 0.56%). As expected, SA greatly inhibited the apoptosis in both H_2_O_2_-induced and non-induced cells (Figure 3H,G). The apoptosis rates were reduced to 2.17% ± 0.20% and 3.1% ± 0.34%, respectively, after treatment with SA alone or SA/H_2_O_2_. Differences were significant (*p* < 0.05) compared with the control group and to the H_2_O_2_ group (*p* < 0.01). SA showed stronger anti-apoptosis effects than resveratrol in the experiments. Apoptosis is a genetically controlled and evolutionarily conserved form of cell death that contributes to the normal embryonic development and the maintenance of tissue homeostasis in the adult organism [16]. It occurs in multicellular organisms and can be initiated by two pathways, i.e., an intrinsic pathway and an extrinsic pathway, both of which depend on the activation of caspases [17]. SA reduced the apoptosis of HUVECs by resisting oxidative stress, precisely by the activation of the intrinsic pathway, which has been confirmed by the robust inhibiting effect of SA on the overproduction of ROS induced by H_2_O_2_. Apoptosis is desired in cancer treatment. However, excess apoptosis can lead to neurodegenerative diseases, hematologic diseases and tissue damage, which can be found in myocardial ischemia/ischemia reperfusion injury and HIV infection. Anti-apoptotic drugs, such as nerve growth factor and antioxidant, have been proved to be effective for the prevention and amelioration of these pathological conditions. Protocatechuic acid has been reported to be effective in preventing neurodegenerative diseases such as Parkinson’s disease in PC12 cell model, by virtue of its prominent antioxidative, anti-cytotoxic and anti-apoptotic activities [18,19]. Taking into account all these findings, SA might be a promising molecule for injury recovery and degenerative diseases due to its mighty anti-apoptotic activity. 

### 2.3. ROS Detection Assays

**Figure 4:**
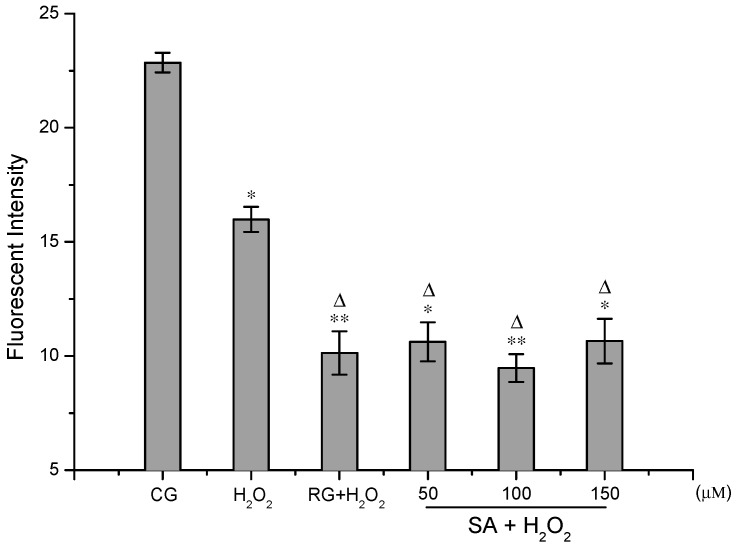
Results of ROS detection assays. CG represents as control group. RG represents as positive control group. H_2_O_2_ represents as the group treated with H_2_O_2_ alone. Values are means ±SD (*n* = 3). Compared with the control group (* *p* < 0.05, ** *p* < 0.01). Compared with the H_2_O_2_ group (^∆^ *p* < 0.05).

The overproduction of the intracellular ROS in HUVECs was induced by H_2_O_2_. Figure 4 demonstrated the inhibition effect of SA on the generation of ROS. The ROS levels were lessened after the cells were treated with SA or resveratrol, and there were significant differences at 1% or 5% level compared with the control and H_2_O_2_ groups. However, there was no distinct dose-response relationship between SA and ROS levels at concentrations from 50 to 150 μM. After pre-incubated with 100 μM of SA, the fluorescent intensity in the cells was abated to 9.47 ± 0.61, which was much less than the control group (22.85 ± 0.43). SA remarkably crippled the ROS levels in HUVECs. Oxidative stress has been linked with the pathogenesis of several degenerative diseases including aging, atherosclerosis, cardiovascular diseases, neurodegenerative diseases and cancer [16]. ROS in the form of superoxide anion radical (O_2_^−•^), hydroxyl radical (^•^OH), hydrogen peroxide (H_2_O_2_), singlet oxygen (^1^O_2_), and nitric oxide (NO^•^) are highly reactive species and their uncontrolled generation induces oxidative modification of cellular macromolecules, which can ultimately lead to apoptotic or necrotic cell death [20,21]. In this regard, the inhibitory effect of SA on ROS generation in HUVECs acts as one of the major mechanisms for SA-mediated cytoprotection. The intracellular ROS are generated by the normal metabolic process or from exogenous factors and agents, and they can be scavenged by endogenous antioxidative enzymes including catalase, superoxide dismutase and heme oxygenase, signaling pathways and exogenous antioxidants. Berberine hydrochloride (BBH) could protect C2C12 cells against H_2_O_2_-induced oxidative stress via suppression of the accumulation of intracellular ROS, which involved the activation of the Nrf2/HO-1 pathway [22]. Astaxanthin was able to protect *C. elegans* from oxidative damage potentially by modulating genes involved in the insulin/insulin-like growth factor (IGF) signaling (IIS) pathway and the oxidoreductase system [23]. In view of these results, the molecular mechanisms for ROS inhibition can be considered so sophisticated that further studies are required to elucidate SA-mediated mechanisms in detail.

## 3. Materials and Methods

### 3.1. Materials

SA was obtained from *Athyrium multidentatum* (Doll.) Ching rhizome, which was collected in Changbai Mountain area of China in 2012 and identified by Professor Chongmei Xu (Department of Pharmacy, Weifang Medical University). Fetal bovine serum (FBS) was purchased from Sijiqing Biological Engineering Materials Co., Ltd (Hangzhou, China). Hyclone RPMI 1640 medium, phosphate buffered saline (PBS), penicillin-streptomycin, MTT cell proliferation and cytotoxicity assay kit, Annexin V-FITC/PI apoptosis detection kit and dimethyl sulphoxide (DMSO) were from Solarbio^®^ Science & Technology (Beijing, China). Reactive oxygen species assay kit was purchased from Beyotime^®^ Biotechnology (Shanghai, China). Resveratrol was from Sinopharm Chemical Reagent Co., Ltd (Shanghai, China).

### 3.2. Preparation of Samples

SA was prepared as described in our previous study [10]. In brief, the dried rhizome of *Athyrium multidentatum* (Doll.) Ching was finely chipped and soaked in methanol at room temperature for 20 days. The resultant extract was suspended in distilled water and partitioned successively with petroleum ether and ethyl acetate. The ethyl acetate extract was further eluted and purified by silica gel column chromatography (100–200 mesh) eluting with petroleum ether and ethyl acetate. SA was obtained from the fraction that eluted with 4:1 (*v*:*v*) petroleum ether and ethyl acetate. SA redissolved in DMSO was normalized to a final concentration of 0.2% DMSO in culture medium when applied to the cells.

### 3.3. Cell Culture

The HUVECs were obtained from Institute of Plastic Surgery, Weifang Medical University (Weifang, China). Cells were maintained in RPMI-1640 medium supplemented with 10% heat-inactivated FBS and 1% antibiotics in a water-saturated atmosphere of 5% CO_2_ at 37 °C. The culture medium was changed every other day and all the operations were processed under sterile conditions. Cells of passage 3–5 were used in all experiments. 

### 3.4. Analysis of Cell Viability

The MTT assay was conducted to evaluate the cell proliferation and protection effects of SA against H_2_O_2_-induced oxidative damage [24]. In the cell proliferation assay, HUVECs were plated at 100 µL of 5 × 10^3^ cells per well in a 96 well plate. Following stabilisation for 12 h, the cells were treated with 100 µL different concentrations of SA (50–300 µM) or resveratrol (20 µM). Meanwhile, the control group (CG) was added 100 µL of 1640 growth medium instead of medicine. The group without cells was designed as vacuity control group. After 48 h of incubation, cells were washed two times with PBS, then 90 µL fresh 1640 growth medium and 10 µL MTT reagent (5 mg/mL) were added sequentially for the formation of formazan. Four hours later, 110 µL of DMSO was added and gently shaken for 10 min to dissolve the formazan crystals. Finally, the absorbance was read at 570 nm using a microplate reader (Thermo Scientific, Waltham, MA, USA). The best non-toxic sample concentration (over 100% cell viability) in the cell proliferation was selected for cell apoptosis evaluation assay. 

The cytoprotective effect of SA against H_2_O_2_-induced oxdative injury was performed as a modified method of Guan et al. [25]. Briefly, after digested by 0.25% trypsin, HUVECs were adjusted to the concentration of 1 × 10^4^ cells/mL and inoculated into a 96-well cell culture plate (100 μL per well), then cultured at 37 °C for 12 h. Afterwards, cells were treated with SA (50–300 µM) or resveratrol (20 µM) for 48 h before co-incubated with 300 µM H_2_O_2_ solution for 40 min. At the end of treatment, cells were washed once with PBS and the cell viability was measured as described previously. Cell proliferation and protective effects by the samples were expressed as the percentage of cell viability, and calculated according to the following formula:

Cell viability (%) = (*OD*_sample 570_ − *OD*_vacuity 570_)/(*OD*_control 570_ − *OD*_vacuity 570_) × 100, (1)

### 3.5. Cell Apoptosis Assays

Apoptosis in HUVECs was analyzed by flow cytometry with the Annexin V-FITC/PI apoptosis kit according to the manufacturer's protocols. Cells were exposed to SA (100 µM) or resveratrol (10 µM) for 48 h, then washed twice with PBS. About 1 × 10^6^ cells (per well) were harvested by centrifugation at 900 rpm for 3 min, successively washed once with cold PBS and binding buffer, then resuspended with 500 µL binding buffer. 5 µL Annexin V-FITC was added into the cells (100 µL) and reacted in the dark for 10 min. Afterwards, 5 µL PI was added and incubated for another 5 min. Finally, 300 µL PBS was added and the cell apoptosis was analyzed on a flow cytometer (BD FACSCalibur, Anaheim, CA, USA). 

Apoptosis in H_2_O_2_-treated HUVECs was determined by the same method. Briefly, after treatment with SA (100 µM) or resveratrol (10 µM) for 48 h, cells were added 500 µL H_2_O_2_ (300 µM final concentration) solution and endured for 40 min, then washed twice with PBS. After stained with Annexin V-FITC and PI, the cells were applied to the analysis of apoptosis. 

### 3.6. ROS Detection Assays

The intracellular ROS levels were measured according to a modified method of Yang et al. [26]. About 7 × 10^5^ cells (per well) were plated into a 6-well plate and allowed to stabilize for 12 h, then treated with SA (50–150 µM) or resveratrol (10 µM) for 48 h. At the end of treatment, 0.5 mL H_2_O_2_ solution (300 µM final concentration) was added to induce the overproduction of ROS. 40 min later, the cells were washed with PBS and collected by centrifugation at 900 rpm for 3 min, then incubated in the dark with 10 µM DCFH-DA prepared in serum-free media (1:1000). After incubation for 20 min at 37 °C, the cells were washed three times with FBS-free 1640 medium and transferred to a 96-well black plate. Cellular fluorescence was measured using a Fluoroskan Ascent fluorometer (Thermo Scientific). Reading was taken at excitation and emission wavelengths of 485 and 538 nm, respectively. Results were expressed directly as fluorescent intensity.

### 3.7. Statistical Analysis

Data were presented as means ± SD from three independent experiments and evaluated using the statistical software of Microsoft Excel 2003 coupled with a two-tailed paired t-test. Statistical significance was considered significant if *p* < 0.05.

## 4. Conclusions

The results clearly demonstrated that SA exhibited excellent proliferative and slight protective effects on HUVECs through a combinination of anti-apoptosis and ROS inhibition actions. The current findings give support for the potential application of SA in the prevention of degenerative diseases and the development of protective ingredients against oxidative damage.
